# The Influence of Posterior Class II Composite Restoration Location and Techniques on Marginal Sealing

**DOI:** 10.3390/dj13010039

**Published:** 2025-01-17

**Authors:** Mishel Haddad, Diva Lugassy, Mohana Barhum, Tamar Brosh, Shlomo Matalon

**Affiliations:** 1 Department of Oral Rehabilitation, The Maurice and Gabriela Goldschleger School of Dental Medicine, Faculty of Medicine, Tel Aviv University, Tel Aviv 6997801, Israel; mohanabarhum@mail.tau.ac.il (M.B.); matalons@tauex.tau.ac.il (S.M.); 2 Department of Orthodontics, The Maurice and Gabriela Goldschleger School of Dental Medicine, Faculty of Medicine, Tel Aviv University, Tel Aviv 6997801, Israel; divaluga@mail.tau.ac.il; 3 Department of Oral Biology, The Maurice and Gabriela Goldschleger School of Dental Medicine, Faculty of Medicine, Tel Aviv University, Tel Aviv 6997801, Israel; tbrosh@tauex.tau.ac.il

**Keywords:** resin composite, Class II, Pre-cure, Co-cure, gap formation

## Abstract

**Background/Objectives**: The success of treatment and prevention for secondary caries hinges significantly on the techniques employed in Class II composite restoration. Additionally, the location of the restored tooth within the oral cavity has emerged as a potential factor determining the quality of the restoration. A comprehensive understanding of these interrelated variables is crucial for advancing the efficacy and durability of dental composite restorations. The aim of this study was to assess how various restoration techniques and the specific location of the tooth restoration in the oral cavity affect marginal sealing, verified by the gap created in the tooth–restoration interface. **Methods**: Sixty extracted human teeth that had been indicated for orthodontic extraction were collected and embedded into a custom-made holder that was located in one of the four quadrants of a laboratory phantom head. Class II resin composite restorations, using flowable and packable composites, were performed on all teeth using two techniques: Pre-cure and Co-cure. The aging of the restored teeth was conducted using cyclic loading and thermocycling. After aging, the teeth were examined under a scanning electron microscope to measure the gap within the tooth–composite interface. Kolmogorov–Smirnov tests were used to assess the data distribution. Unpaired *T*-tests were employed to compare the mean gaps between the Pre-cure and Co-cure techniques. Additionally, unpaired *T*-tests were utilized to compare the mean gaps between the mesial and distal parts of the teeth. The Kruskal–Wallis test was used to compare the mean gaps among the four quadrants. The statistical significance was set at *p* = 0.05. **Results**: No significant difference in the gaps between the Pre-cure and Co-cure techniques was found (*p* = 0.212). The tooth’s location did not generally affect the restoration’s gap interface (*p* = 0.136). **Conclusions**: Flowable composites aid in restoring the deep margins of Class II composite restoration. Thus, the potential for further microleakage is similar for both the Pre-cure and Co-cure restoration techniques. The marginal seal of Class II composite restorations is effective when using both Pre-cure and Co-cure techniques, and the restoration site within the oral cavity does not significantly influence the tooth–composite interface seal.

## 1. Introduction

Dental composites, a tooth-colored restorative material, were first introduced in the mid-1950s. Its popularity stems from its esthetic appeal, conservative restoration approach, and ease of manipulation, making it the preferred choice for Class I and Class II posterior restorations [1]. For Class I and Class II posterior restorations, conventional composites with higher filler contents are often used due to their stress-bearing properties. This results in high strength and resistance and an amalgam-like handling experience in the proximal box of Class II restorations. Due to the increased filler content, posterior restorations benefit from elevated wear resistance and minimal polymerization shrinkage [2,3]. Flowable composites have a reduced filler content (50–70%/wt) but the same particle size as traditional composites. This reduced filler content decreases the viscosity and enhances the flowability of the composite. Despite differences in their relative flow and radiopacity, flowable materials have comparable strength, with approximately half the rigidity of normal composites and approximately 80% of the flexural strength [4,5].

Flowable dental composites have low viscosity and high adaptability in restorations with a filler volume that is reduced by 30% compared to conventional composites. This low viscosity improves the manipulation quality and enables simple syringe dispensing to be performed [6]. Flowable resin composites are used in a variety of applications, including pit and fissure sealers, minimal restorative techniques, cavity liners, and class V restorations. The average polymerization shrinkage of flowable composites is up to 5% [7]. High resin matrices with fewer fillers may cause greater polymerization shrinkage than conventional or compact composites. However, a low elastic modulus provides a better seal, fracture resistance, and durability as a lining material in the majority of posterior restorations [8].

The most common restoration failures in resin-based restoration are secondary caries or fracturing. Marginal integrity and bonding significantly impact the longevity of restorations, especially in Class II restorations, where thin enamel surfaces can lead to marginal ditching and leakage [9,10]. Microleakage, the passage of substances between the tooth and restoration, can lead to the failure of the restoration [11].

Previous studies have compared the Pre-cure and Co-cure techniques employed in laboratory studies [12,13]. However, as flowable composites have lower viscosity, it is thought that the location of the tooth restoration within the jaw in a clinical situation could affect the restoration’s quality.

Nevertheless, researchers have investigated artificial marginal leakage by examining the gap that forms between restorations and the tooth structure after aging. Aging introduces fatigue at the adhesive interface through repeated mechanical stress [14,15,16,17,18,19,20]. To mimic clinical conditions, which include various food temperatures and chewing, in a laboratory, restorations are commonly subjected to aging processes via thermocycling and cyclic loading. A review study noted that thermocycling cycles range from 500 to 100,000 at water temperatures of 5–55 °C in most cases [21]. Another review study stated that the number of cycling loading cycles is arbitrary and ranges between 1000 and 50,000 [20]. To evaluate the bonding quality after aging, one approach measures bond strength using a loading machine [14]. Another method involves sectioning the restored tooth and evaluating the gap formed via dye penetration [13]. Some studies have also created gap replicas using polyvinylsiloxane impressions and analyzed their thickness using an SEM [18].

Direct dental procedures, such as resin composite restorations, require high-level clinical skills, knowledge, and experience to deal with the different factors affecting the marginal seal of the restoration, such as the type of material used, the method of restoration, and the location of the restored tooth in the oral cavity, along with operator-related factors [19]. Clinical studies show that Class II restorations have a lower survival rate than Class I restorations [22]. Their location in the oral cavity affects the survival of the restored tooth. Some studies have found that the survival rate of maxillary restorations is higher than that for mandible restorations [23,24,25], while some have found that mandibular restorations have greater longevity [26,27]. 

Nevertheless, it can be assumed that it is not only whether the restoration is located in the maxilla or mandible that affects the quality and survival of the restoration but also the application of Class II restorations in mesial vs. distal procedures. However, in vitro experiments have not considered the location of the tooth when preparing restorations in the lab, and restorations have primarily been performed on a laboratory bench.

Various restorative procedures have been used to achieve a better marginal seal for composite restorations. Flowable composites can be used as a liner in Class II cavities with varying lining thicknesses. The two major lining processes are Co-cure and Pre-cure polymerization [28].

The Co-cure technique enables the simultaneous curing of flowable and regular composites in a single phase, while the Pre-cure technique involves the curing of the flowable composite before compact composite curing. In Class II restoration, both techniques have different effects on adhesion and the marginal seal. Most manufacturers suggest that a pre-curing process for flowable composites is used in order to obtain a greater marginal seal [29,30]. However, little research regarding the use of an uncured mixture of flowable and conventional resin composites, as observed in the Co-cure method, has been published [6,31].

One crucial factor to consider when striving for optimal outcomes is the positioning of the restored tooth within the oral cavity. This can be influenced by various factors, including the gravitational impact experienced in the maxillary arch versus the mandibular jaw [19]. This distinction is particularly relevant when working with flowable restoration materials, as it can significantly impact the durability of the restoration over time. Previous studies have predominantly focused on specific aspects of dental composite restorations, yet the impact of the tooth’s location within the oral cavity has received limited attention. This study examined restorations in all four quadrants to assess the potential differences between the maxilla and mandible, considering the influence of gravity on the flowable composite. It also aimed to test two null hypotheses. First, it assumed that both restoration techniques (Co-cure and Pre-cure) would have comparable effects on the gap that emerges between the restoration and the tooth following the aging process. Second, it hypothesized that the location of the tooth’s restoration within the oral cavity, along with whether the restoration was situated in the mesial or distal area of the tooth, would not affect this gap.

This laboratory study aimed to address the following research question: How do different restoration techniques and the specific location of the restoration within the oral cavity affect the restoration’s quality? The primary outcome of interest was the gap within the tooth–composite interface, which is indicative of the quality of the marginal seal.

## 2. Materials and Methods

Sixty human caries-free premolar teeth that had been indicated for extraction for orthodontic purposes were obtained. The 60 premolars were collected in accordance with the following inclusion criteria: sound premolar teeth indicated for orthodontic extraction and preserved in a physiological solution from the moment of extraction until testing. In total, four out of the sixty teeth were broken during cyclic loading, so the gaps for these teeth were not considered (93% survival).

All teeth were collected according to a consent form that was signed by all patients receiving treatment in the Dental School and approved by the ethics committee (no. 0007546-1 approved on 15 November 2023).

### Teeth Holder

**Figure 1:**
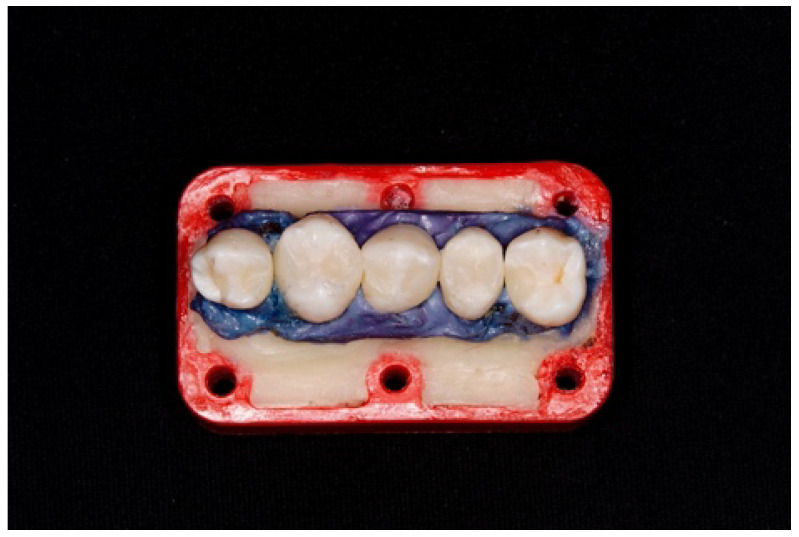
Five teeth embedded in the plastic holder.

**Figure 2:**
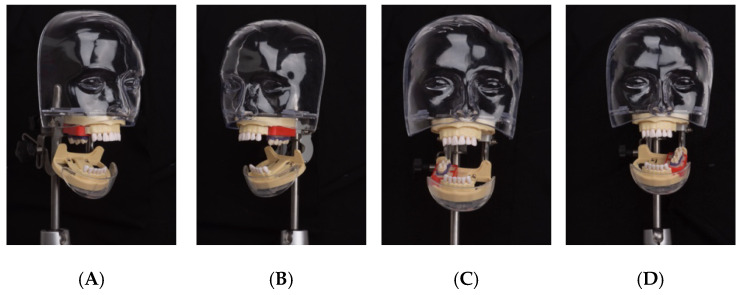
Holders with embedded teeth were placed in the posterior area of the four quadrants of the phantom head: (**A**) upper right; (**B**) upper left; (**C**) lower right; (**D**) lower left. The phantom head was placed in a position simulating the patient’s position relative to the dentist. Then, each proximal side of the teeth was restored using either technique, except for the extremities; that is, there were eight restorations in each holder.

Custom plastic holders were fabricated to fit securely in the posterior areas of each of the four quadrants in the phantom head in order to simulate the dentist–patient position during teeth preparations and restorations. This had not been undertaken previously (Figure 1 and Figure 2). Each holder was designed to accommodate five teeth. To create a 0.25 mm space for simulating the periodontal ligament, each tooth was initially wrapped in dental wax. This space would later enable the injection of the Impregum material. Acrylic dental resin (UNIFAST TRAD GC America, Alsip, IL, USA), used to represent the bone, was first injected into each holder. The wax-wrapped teeth were temporarily inserted into the partially set acrylic resin and subsequently removed before the resin fully hardened. Then, the wax was removed from each tooth via immersion in hot water. Once the acrylic resin had set, Impregum soft impression material (3M; Neuss, Germany), simulating the periodontal ligament, was injected into the spaces created by the teeth within the acrylic material. The teeth were then reinserted into the Impregum-filled spaces until the material was set [32].

The 12 holders were randomly divided into four groups, with each group assigned to one of the four quadrants (upper left, upper right, lower left, and lower right) of the phantom head (Figure 2).

The phantom head was positioned to simulate the clinical conditions [33], being parallel to the ground for the upper arch and at a 45-degree angle for the lower arch. All procedures were performed using indirect vision, closely mimicking the posture of a patient in a dental chair. The task of performing cavity preparations and restorations was carried out exclusively by one operator (M.H.), who had 8 years of clinical experience.

Class II cavity preparations were performed using a B2 diamond bur (Strauss & Co., Ra’anana, Israel).

For each of the three central teeth in each holder, two cavities were prepared: one on the mesial and one on the distal surface. The two outermost teeth, however, received only one cavity preparation each; this was mesial for the most distal tooth and distal for the most mesial tooth. This setup was designed to simulate realistic Class II restorations in which adjacent teeth provide proximal support. Since the outermost surfaces of the end teeth lacked adjacent teeth, these surfaces were left unrestored.

It is worth noting that the base of the gingival floor was strategically positioned 1 mm above the cementoenamel junction (CEJ), reflecting a key anatomical reference point.

A circumferential metal matrix (Kerr SuperMat Matrix System, Kerr, Brea, CA, USA) was placed around the tooth, and cavities were etched with a selective etching technique for 15 s using 37% phosphoric acid etching gel; the teeth were then rinsed with water for 15 s and gently dried.

GC G-Premio-Bond universal bonding (GC Corporation, Tokyo, Japan) was used following the manufacturer’s instructions. The bonding material was applied to the entire cavity using a disposable applicator and rubbed in for 20 s; then, a gentle stream of air was applied until the material ceased moving, indicating that the solvent had evaporated completely. The bonding agent was cured using an LED curing light (Guilin Woodpecker, Guilin, China 1250 mW/cm^2^) for 20 s.

Three teeth were adjacent to the additional tooth. Each aspect of these teeth was restored using a different technique: half of the total occluso-mesial cavities were restored using the Pre-cure technique, while the remainder were restored using the Co-cure technique. The same was performed for occluso-distal cavities.

The most mesial and distal teeth in each holder were restored using one of the techniques. 

By restoring each tooth using a different procedure, the effect of differences between the mesial and distal aspects of the teeth could be considered.

This setup yielded 4 restorations in each of the 3 holders, resulting in 12 restorations using the Pre-cure technique and 12 restorations using the Co-cure technique in each quadrant.

The Co-cure restoration technique involved several steps. Initially, a thin layer of flowable composite (G-aenial Universal Flo flowable; GC Corporation, Tokyo, Japan) was applied over the gingival floor of the prepared cavity. Following this, a horizontal increment of packable resin composite (Gradia Direct; GC Corporation, Tokyo, Japan) was applied using a condenser. The layered flowable composite and restorative resin composite were then cured using an LED curing unit (Guilin Woodpecker; Guilin) for 20 s. The remaining portion of the cavity was restored using two horizontal increments of packable resin composite (Gradia Direct; GC Corporation, Tokyo, Japan). Each increment was light-cured for 20 s, followed by a final curing period of 40 s.

In the Pre-cure restoration technique, a thin layer of flowable composite (G-aenial Universal Flo; GC Corporation, Tokyo, Japan) was initially placed over the gingival floor of the prepared cavity. This layer was light-cured for 20 s. Subsequently, the rest of the cavity was restored using three horizontal increments of packable resin composite using a condenser (Gradia Direct; GC Corporation, Tokyo, Japan). Each increment was light-cured for 20 s, followed by a final curing period of 40 s.

After restoration, any excess composite in the gingival area was eliminated using a surgical scalpel blade (#12).

**Figure 3:**
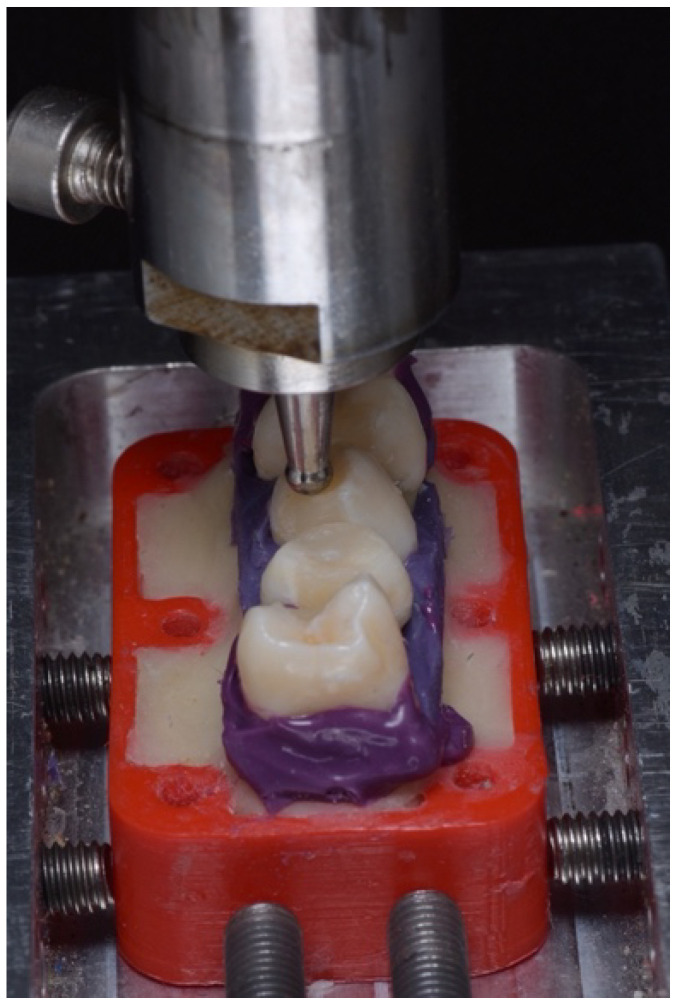
Setup of the cyclic loading procedure. Each of the five teeth within the holder was individually placed under the tip of the cyclic loading machine one at a time (the wet gauze covering the unloaded teeth, applied to prevent dehydration, was removed for imaging purposes).

The restored teeth underwent aging procedures. A cyclic loading machine (J. MANAS LTD, Holon, Israel) was used to apply 20,000 cycles of 100 N force at the central fossa of each tooth. To prevent dehydration during this process, the rest of the teeth within the same holder were wrapped with dental gauze soaked in water (Figure 3).

Subsequently, all teeth were subjected to thermocycling (J. MANAS LTD, Holon, Israel), undergoing 2000 cycles between 5 °C and 55 °C, with a 20 s dwell time for each temperature.

**Figure 4:**
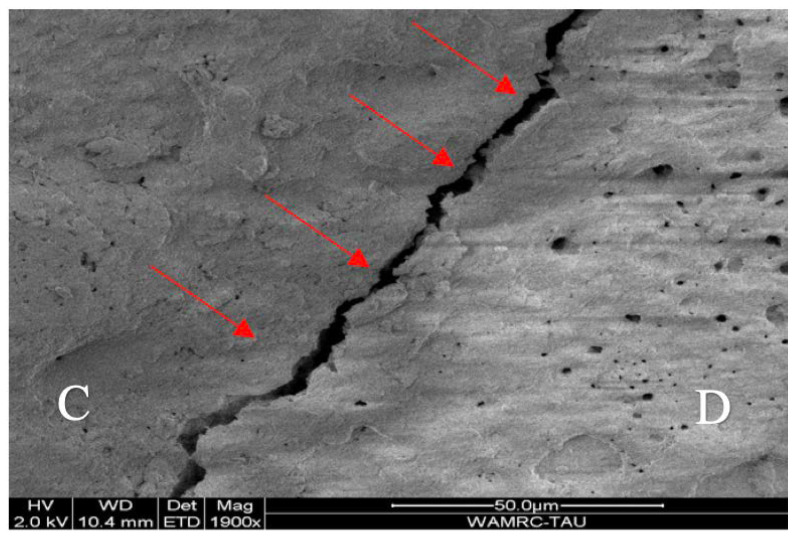
Gap between composite (C) and dentine (D) under a scanning electron microscope (SEM).

After aging, the teeth were removed from the holder and coated with a thin layer of chromium (Cr) via vapor deposition in an argon gas atmosphere using a sputtering machine (Quorum SC7620 Mini Sputter Coater, Quorumtech, Lewes, UK); this was performed to improve the conductivity and image quality. The gaps were examined under scanning electron microscopy at a magnification of X1900 (JSM-IT 100 InTouchScop SEM (JEOL, Tokyo, Japan). The operator (M.H) was blinded to the experimental groups under the microscope, as the samples were placed under the microscope by another individual. When examining the gaps, the operator was solely responsible for reporting the measurements, ensuring that he was unaware of the specific details of the samples being analyzed. For each tooth, the gaps at the tooth–restoration interface were recorded (Figure 4). The gingival interface was divided into three segments with a size of approximately 0.6 mm. The three largest observed gaps were measured in microns (μm) for each segment, resulting in nine measurements for each restoration. The mean value of these nine measurements defined the gap size for each restoration. 

Table 1 presents the steps used to perform the restorations, aging, and examination under an SEM.

Kolmogorov–Smirnov tests were used to assess the data distribution. Unpaired *T*-tests were employed to compare the gaps observed when the Pre-cure and Co-cure techniques were employed. Additionally, unpaired T-tests were utilized to compare the gaps between the mesial and distal aspects of the teeth. The Kruskal–Wallis test was used to compare the gaps among the four quadrants. Sensitivity power analysis using G*Power was employed to determine which effect size was sensitive enough for detection in this study. Statistical significance was set at *p* = 0.05.

## 3. Results

To compare the restoration techniques and mesio-distal surfaces, an independent *T*-test with 48 restorations was used to detect the effect size of Cohen’s f = 0.59 with 80% power (α = 0.05). This corresponds to a medium effect size.

In order to compare the quadrants, a Kruskal–Wallis test with 24 restorations was used to detect the effect size of Cohen’s f = 0.84 with 80% power (α = 0.05). This corresponds to a high effect size. Table 2 presents the mean gaps, measured in microns, when both the Pre-cure and Co-cure techniques were used; it also shows both tooth aspects, distal and mesial, of the gingival margin. The mean gaps were 4.197 ± 1.838 µm and 4.035 ± 1.503 µm for the Pre-cure and Co-cure techniques, respectively, with no statistically significant differences between them (*p* > 0.212). The mean gaps were 4.098 ± 1.479 µm and 4.140 ± 1.890 µm for the mesial and distal aspects, respectively, with no significant differences between them (*p* > 0.443).

**Figure 5:**
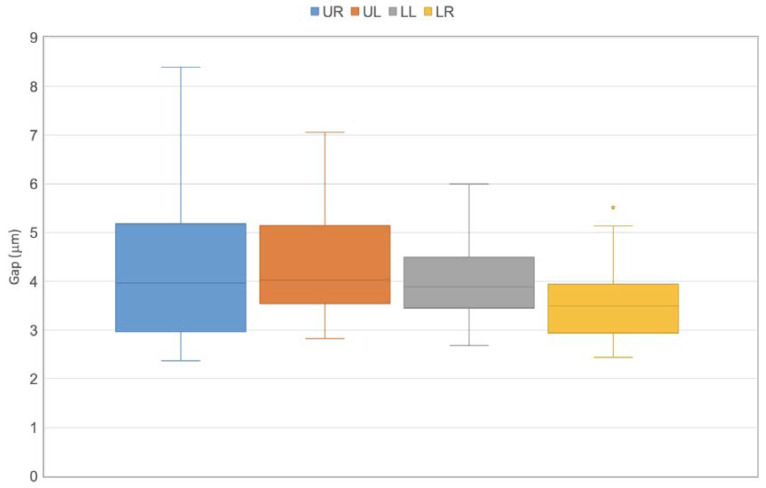
Distribution of gap measurements for the four quadrants when the Pre-cure and Co-cure techniques were employed.

A box diagram representing the gaps, measured in microns, for the four quadrants is presented in Figure 5. The mean gaps for the upper right, upper left, lower left, and lower right were 4.232 + 1.717 µm, 4.696 + 2.311 µm, 4 µm + 1.158, and 3.591 + 1.197 µm, respectively, with no statistically significant differences between the quadrants (*p* > 0.136). Interestingly, both the mean gaps at the maxilla and the standard deviation for that jaw were higher than in the mandible.

## 4. Discussion

The null hypotheses of the current study were approved: No significant difference, in terms of gaps, was found between the Pre-cure and Co-cure techniques and the restored tooth after aging via cyclic loading and thermocycling. The location of the restored tooth in the oral cavity and the aspects of the tooth also had no significant effect on the gap formed.

Most laboratory studies do not simulate the position of the oral cavity during clinical treatment. In this study, not only was the patient’s clinical position simulated, but a method described by Silva et al. and used to replicate the bone and periodontal ligament surrounding natural teeth was also simulated [32]. It was hypothesized that the viscosity of the restorative materials used, along with the location of the restoration within the jaws, might influence the long-term quality of the restoration after aging. This hypothesis arose from the differing positions of the teeth in the upper and lower jaws. Specifically, maxillary teeth might be more affected by gravity during the restorative process, as the flowable composite material is injected in a direction that is against gravity. In contrast, mandibular teeth, being positioned lower in the mouth, may not experience the same gravitational forces, potentially leading to differences in the quality of the restoration between the upper and lower jaws. However, in this study, no significant differences in gaps between the restorations and the teeth were observed across the four quadrants. Previous studies have been inconclusive when comparing the efficacy of Pre-cure and Co-cure restoration techniques. Chuang et al. showed that, when using the Co-cure technique, the flowable liner and the overlaying composite enable the uncured liner to penetrate more deeply and improve the seal at the margin; this is due to the hydraulic pressure of the higher-viscosity composite overlying it [12]. Other studies suggested that better marginal sealing could be achieved by using the Pre-cure technique [13,34].

An important aspect of posterior teeth restoration is the adaptability of the material used in the cervical area. It is recommended that flowable composites are used to enhance the adaptation of the viscous material, especially in proximal boxes. The use of flowable composites as a liner in composite restoration has various advantages. The flowable composites are first placed in the cavity using a syringe, enabling the smooth flow of the preparation. This can aid the operator in covering the entire preparation more easily. It can be assumed that this more precise insertion procedure lowers the likelihood of voids occurring. Secondly, the flowable composite liner may operate as a flexible intermediate layer, relieving tensions during the polymerization shrinkage of the restorative resin. This is due to the low Young’s Modulus of flowable composites compared to other hybrid composites. This could help to dissipate contraction stresses produced by polymerization.

The flowable composite only shows excellent properties in the contact-free area that has a thickness of 1 mm in the gingival wall. The flowable composite has low viscosity, with a low filler content. The placement of an intermediate layer of flowable composite improved the marginal sealing of the restoration and reduced the contraction stress generated by a subsequent layer of high-modulus composite material [30].

In the current study, one clinician performed the clinical procedure. While some clinical studies on posterior restorations involving multiple operators do not report any association between operator variability and outcome, it has been suggested that dentists may perform surgery with extra precision when they are aware of their involvement in a clinical trial, which may reduce operator-related inconsistencies. However, in this study, the use of a single operator introduces additional considerations, as the operator’s experience level can substantially impact the durability of restorations. Lucarotti et al. observed that both age and experience affect restoration outcomes, with less experienced, recently trained dentists often achieving better long-term success. Thus, while operator variability may be minimized in a controlled setting, the experience of a single operator remains a relevant factor in interpreting and generalizing the study’s findings [19,35].

Clinical studies often consider the location of the tooth in the oral cavity and have found differences in survival between the arches. However, the findings are not conclusive: Some show higher failure rates in the mandible [25,26], while other studies show that the upper jaw is at a higher risk of failure [12,27]. Although no significant differences were observed between the upper and lower jaws in the current study, the maxillary jaw tended to exhibit larger gaps (Figure 5), which may make it more susceptible to bacterial penetration and, consequently, secondary caries. This could help explain the higher failure rates observed in maxillary restorations in certain studies. However, including more restored teeth in each quadrant might have emphasized the differences between the jaws.

Because restoring Class II cavities involves many factors that might affect the quality of the restoration, practitioners should be familiar with both techniques when performing such a procedure.

As the maxilla tended to exhibit more gaps compared to the mandible, further research should test whether different curing methods, restoration techniques, and restored teeth locations could potentially affect the quality of composite restorations.

This study, while employing cyclic loading (20,000 cycles) and thermocycling (2000 cycles), which are equal to approximately 25 days of human masticatory cycles [36,37], is limited by its in vitro design; for example, it does not fully simulate the complexities of the oral environment. According to the literature, restorations fail due to a combination of structural breakdown, surface degradation, and interface loss, caused by the cumulative impact of mechanical, chemical, thermal, and bacteriological stresses in the mouth. These intraoral factors lead to fatigue, a key consideration in evaluating the durability of restorations. Clinical trials are therefore essential in understanding the long-term outcomes, as no single in vitro test can encompass all these degradation patterns; therefore, further clinical studies are needed to validate these findings [38]. The above can be considered as a limitation of the present study.

## 5. Conclusions

While flowable composites might aid in restoring the deep margins of Class II composite restorations, no significant differences in terms of gap formation and potential microleakage were present when curing the flowable liner alone or in combination with a packable composite. However, as restoring Class II cavities is associated with many factors that might affect the quality of the restoration, practitioners should be familiar with both techniques when performing such a procedure. Future research should test whether different curing methods, restoration techniques, materials, and restored teeth locations could potentially affect the quality of composite restorations.

## Figures and Tables

**Table 1:** Steps performed during the restoration, aging, and examination of gaps under SEM.

1. Sixty human caries-free premolar teeth indicated for extraction for orthodontic purposes were obtained.
2. Twelve teeth holders were fabricated to fit securely into the phantom head. Each holder was designed to contain 5 teeth. The 12 holders were divided into 4 groups, resulting in 3 holders for each quadrant.
3. For each holder, the following procedure was carried out: (a) Each tooth was initially wrapped in dental wax to create a 0.25 mm space for the further simulation of the periodontal ligament. (b) Acrylic dental resin (UNIFAST TRAD GC America, Alsip, IL, USA), used to represent the bone, was first injected into the holder. (c) The wax-wrapped teeth were temporarily inserted into the partially set acrylic resin and subsequently removed before the resin was fully hardened, resulting in spaces matching the roots of the teeth within the acrylic resin. (d) Wax was removed from each tooth via immersion in hot water. e) Once the acrylic resin had set, Impregum soft impression material (3M; Neuss, Germany), simulating the periodontal ligament, was injected into the spaces created by the teeth within the acrylic material. (f) The teeth were then reinserted into the spaces filled by the Impregum until it was finally set, while tight contacts between adjacent teeth were manually maintained. (g) At this point, we had 12 holders filled with acrylic and Impregum, with each containing 5 teeth.
4. The 12 holders were randomly divided into four groups, with each group assigned to one of the four quadrants (upper left, upper right, lower left, and lower right) in the phantom head.
5. Eight Class II cavity preparations were performed using a B2 diamond bur: in each of the middle 3 teeth, mesial and distal cavities were prepared; meanwhile, in the 2 outermost teeth, only one cavity was prepared in the side at which an adjacent tooth was presented.
6. Cavities were restored using the Pre-cure and Co-cure techniques.
7. Restored teeth were subjected to two aging procedures: (a) Cyclic loading of 20,000 cycles of 100 N. (b) Thermocycling of 2000 cycles between 5 °C and 55 °C, with a 20 s dwell time for each temperature.
8. After the aging procedure, teeth were removed from the holder then coated with thin chromium (Cr) via vapor deposition in an argon gas.
9. The coated teeth were inserted separately (blinded for the operator) into an SEM so that the gaps in each side of the tooth could be examined.

**Table 2:** Mean (SD) gap results: comparison between Pre-cure and Co-cure techniques and between tooth aspects.

Method	N	Mean (µm)	Std. Deviation (µm)	*p*
Pre-cure	47	4.197	1.838	>0.212
Co-cure	45	4.035	1.503	
Mesial	47	4.098	1.479	>0.443
Distal	45	4.140	1.890	

## Data Availability

The original contributions presented in the study are included in the article. Further inquiries can be directed to the corresponding author/s.
